# Acute Warfarin Toxicity as Initial Manifestation of Metastatic Liver Disease

**DOI:** 10.1155/2016/7389087

**Published:** 2016-03-02

**Authors:** Varalaxmi Bhavani Nannaka, Nihar Jani, Masooma Niazi, Dmitry Lvovsky

**Affiliations:** ^1^Division of Pulmonary and Critical Care Medicine, Bronx Lebanon Hospital Center, Bronx, NY 10457, USA; ^2^Department of Internal Medicine, Bronx Lebanon Hospital Center, Bronx, NY 10457, USA; ^3^Department of Pathology and Histology, Bronx Lebanon Hospital Center, Bronx, NY 10457, USA

## Abstract

Near complete infiltration of the liver secondary to metastasis from the head and neck cancer is a rare occurrence. The prognosis of liver failure associated with malignant infiltration is extremely poor; the survival time of patients is extremely low. We present a case of acute warfarin toxicity as initial manifestation of metastatic liver disease. Our patient is a 64-year-old woman presenting with epigastric pain and discomfort, found to have unrecordable International Normalized Ratio. She rapidly deteriorated with acute respiratory failure requiring mechanical ventilation, profound shock requiring high dose vasopressor infusion, severe coagulopathy, worsening liver enzymes with worsening of lactic acidosis and severe metabolic abnormalities, and refractory to aggressive supportive care and died in less than 48 hours. Autopsy revealed that >90% of the liver was replaced by tumor masses.

## 1. Introduction

The coumarin derivative warfarin, which was licensed in the United States in 1954 as the first human anticoagulant [1], remains the most commonly used oral anticoagulant in North America and the United Kingdom [2, 3]. Warfarin exerts its anticoagulant effect by acting as a vitamin K antagonist and inhibiting the biosynthesis of vitamin K-dependent procoagulant factors II, VII, IX, and X [2–4].

On the basis of a study done in 5077 cases with 99,628 emergency hospitalizations, warfarin was implicated in 33.3% of such Emergency Department (ED) visits [5].

Acute liver failure (ALF) secondary to malignant infiltration of the liver is rare and is diagnosed often only after death. In the era of liver transplantation, it is important to reach a definitive diagnosis and identify the cause because liver transplantation is not indicated if malignant infiltration of the liver is present and alternative therapies may be available.

Our case presents a finding of acute warfarin toxicity as initial manifestation of metastatic liver disease in a patient with stable dose of warfarin for 1.5 years with documented stable International Normalized Ratio (INR) over the same period of time.

## 2. Case Presentation

A 64-year-old woman was brought to ED by a family member for generalized weakness for 10 days associated with epigastric pain and discomfort. Patient also reported having a fall one week prior to her presentation to ED. She noticed to have dark stools and red urine within few days of the fall.

Her medical history was significant for atrial fibrillation on warfarin, COPD, active smoking, and hypertension. Additional history of laryngeal cancer was present, which was treated with radiotherapy and chemotherapy four years ago. As a followup after presenting with hoarseness of voice three years ago, recurrent malignancy was ruled out with vocal cord biopsy. No personal or family history of liver disease was identified. There were no changes to her medications, which included warfarin, amlodipine, metoprolol, aspirin, and atorvastatin.

In ED, patient was tachycardic with a pulse of 160/min, afebrile, and normotensive. The patient had no evidence of acute distress or external injury. She had right subconjunctival hemorrhage, and mucous membranes were dry. Heart examination was significant for tachycardia with no murmurs, rubs, or gallops. Lungs had good bilateral air entry with no wheezing, crackles, or crepitations. Abdominal examination revealed soft but mildly tender epigastrium with normal bowel sounds, whereas rectal examination showed stool mixed with dark blood. She was alert and oriented to time/place/person but appeared slightly lethargic, no focal neurological deficits on neuro examination were found.

Laboratory studies revealed anemia with hemoglobin concentration of 7.2 mg/dL with baseline values around 12 mg/dL less than 3 months ago, leukocytosis with white blood cell count of 15 k/uL, normal platelet count of 232 k/uL, prothrombin time (PT) of 169 seconds, partial thromboplastin time (PTT) of 92 seconds, and unrecordable INR. Chemistry showed prerenal azotemia with blood urea nitrogen levels of 54 mg/dL, creatinine of 1.3 mg/dL, bicarbonate of 10 mEq/L, and normal serum electrolytes. Liver function tests (LFTs) showed hypoalbuminemia with albumin of 2.8 g/dL, transaminitis with alanine aminotransferase (ALT) of 420 U/L, aspartate transaminase (AST) of 973 unit/L, and alkaline phosphatase (ALP) of 435 unit/L. Other significant laboratory values were elevated lactic acid to 13.3 mmoles/L, lactate dehydrogenase (LDH) level of 8003 unit/L, elevated troponin of 0.206 ng/mL, creatine kinase (CK) of 872 unit/L, and creatine kinase MB (CKMB) of 40 ng/mL with MB% of <5. Please refer to Table 1 for laboratory values during the hospitalization. Hepatitis A, B, and C serologies were negative and serum acetaminophen level was <15 ng/dL.

Her INR two weeks prior to her presentation was 2.8 with normal LFTs four weeks prior to admission. She had been on stable dose of warfarin for the past 1.5 years. Please refer to Table 2 for warfarin dosage and INR levels in the past one year.

**Figure 1:**
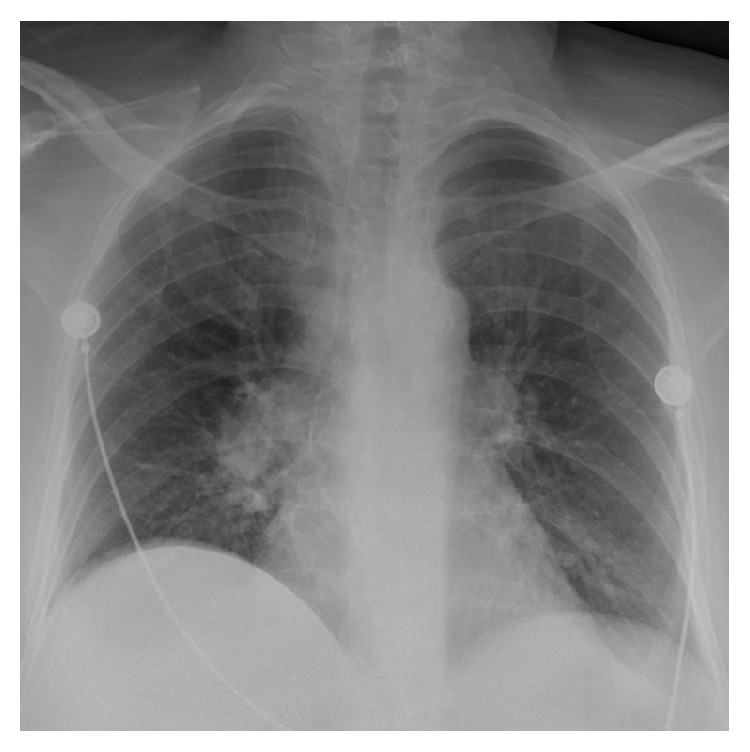
Chest X-ray.

**Figure 2:**
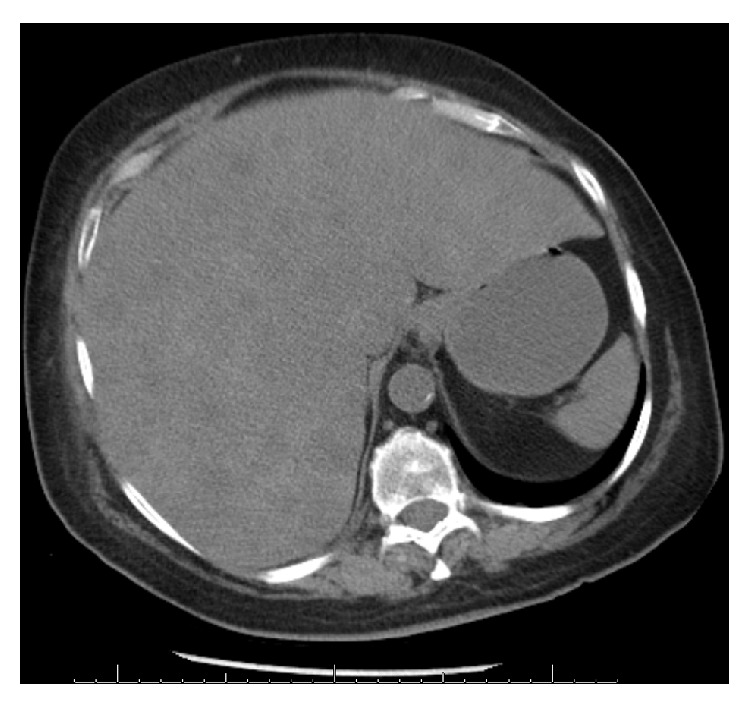
CT of the abdomen without contrast showed markedly enlarged abnormal heterogeneous liver.

Initial chest X-ray (Figure 1) did not show any evidence of an acute pulmonary edema or pneumonia. Computed Tomography (CT) scan of the abdomen (Figure 2) showed markedly enlarged abnormal heterogeneous liver suggestive of an infiltrative process with no obvious free fluid or evidence of significant bleeding. CT scan of the head was negative for acute intracranial hemorrhage, infarction, or masses.

The patient had been given vitamin K and multiple transfusions of Fresh Frozen Plasma (FFP) to reverse coagulopathy.

After admission to intensive care unit (ICU), there was a rapidly progressive decline in the patient's clinical status. She developed acute respiratory failure requiring mechanical ventilation, hypotension necessitating vasoactive agents, and liver failure with worsening LFTs. In addition, she progressed to worsening of coagulopathy, elevated cardiac markers, and lactic acidosis. Her multiorgan failure did not improve with aggressive resuscitative measures, culminating in cardiac arrest and death.

**Figure 3:**
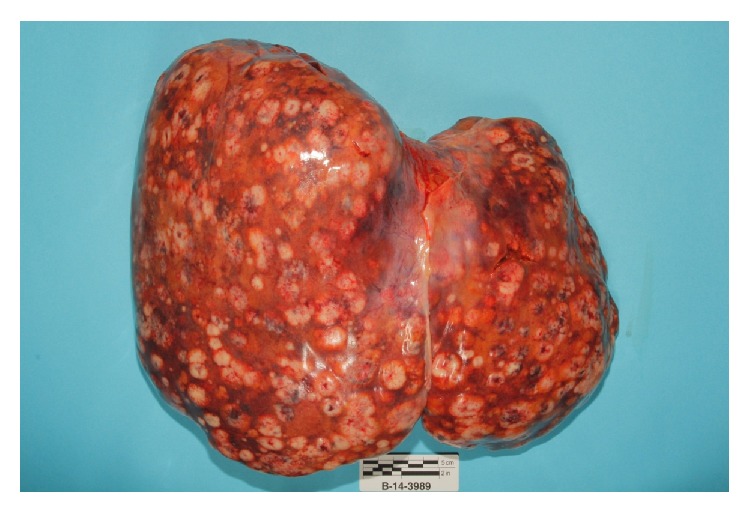
Liver weighed 4950 grams and that is enlarged with intact smooth capsule with soft tan brown parenchyma which was nearly completely replaced by multiple discrete tumor masses.

**Figure 4:**
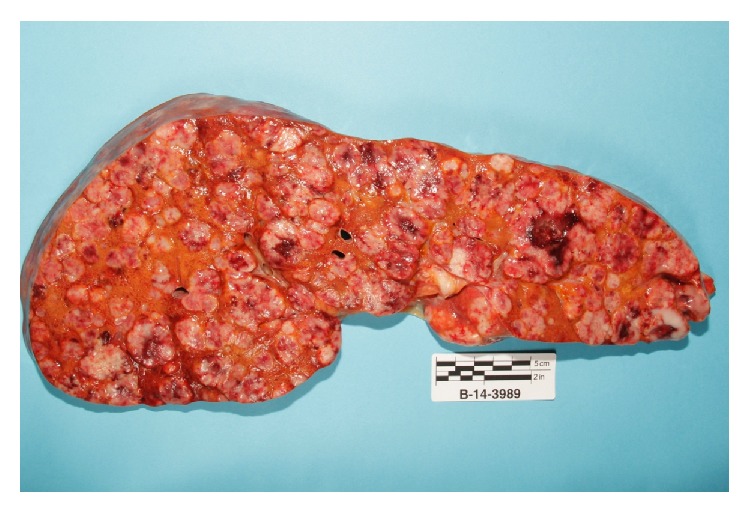
Cut section of liver with diffuse showed involvement by numerous tumor masses and nodules replacing most of the liver parenchyma.

**Figure 5:**
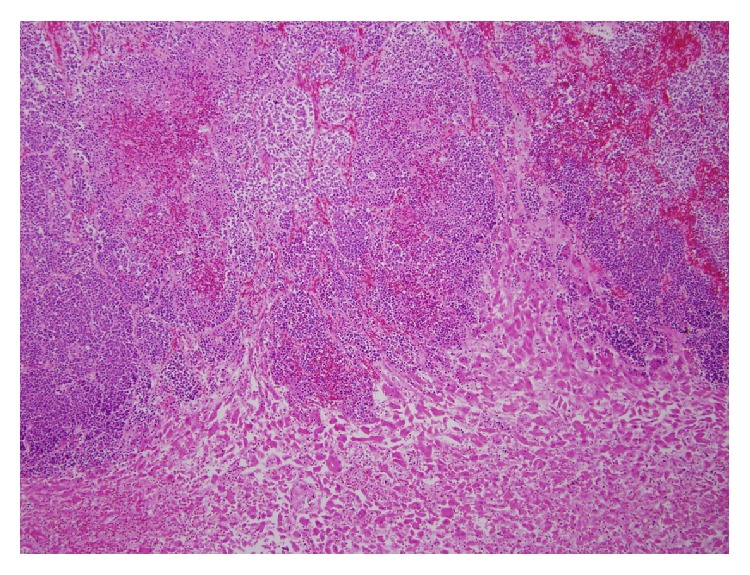
Liver diffusely involved by poorly differentiated squamous cell carcinoma (low power magnification).

**Figure 6:**
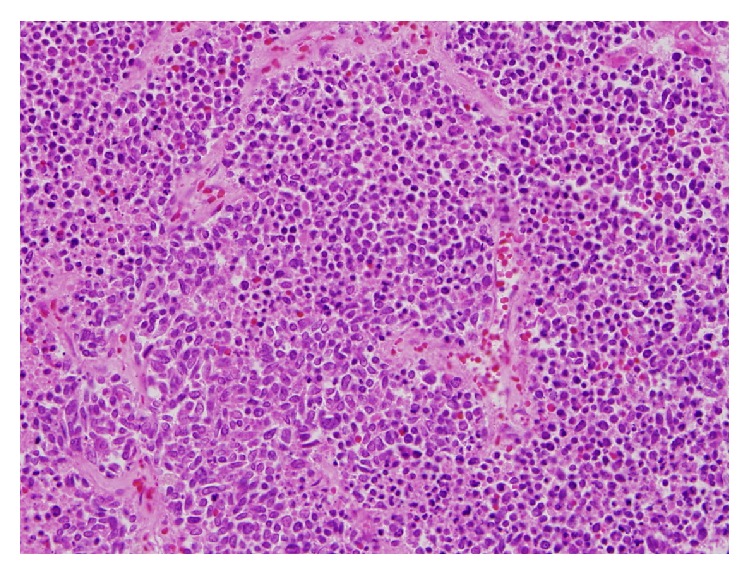
Autopsy of the liver on high magnification showing areas of poorly differentiated squamous cell carcinoma (high power magnification).

**Figure 7:**
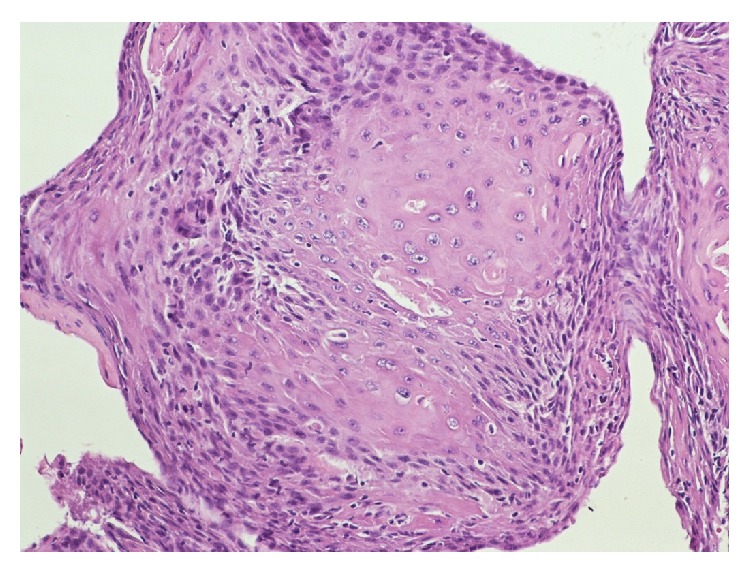
Biopsy of the vocal cord showed invasive well differentiated squamous cell carcinoma showing cohesive nests and intracytoplasmic keratinization.

**Figure 8:**
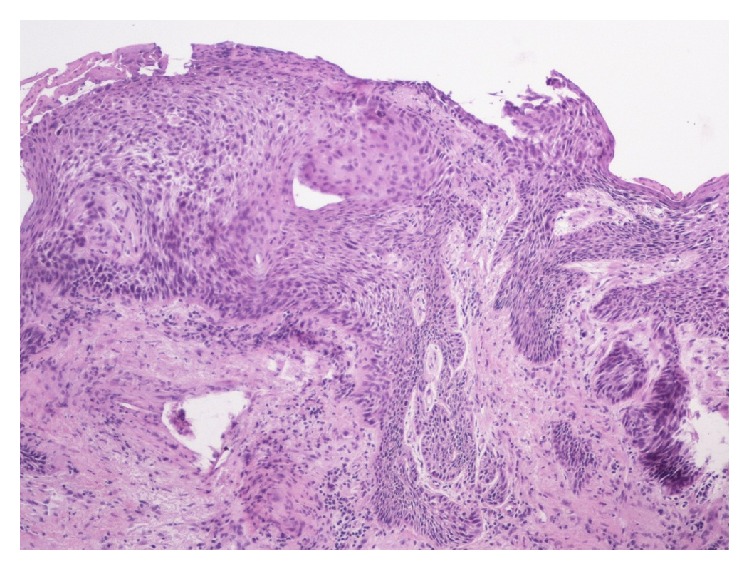
Left vocal cord mass showing deeply invasive, moderately differentiated squamous cell carcinoma.

Autopsy revealed that patient's liver weighed 4950 grams (Figures 3 and 4), and it was enlarged with intact smooth capsule with soft tan brown parenchyma which was nearly completely replaced by multiple discrete and near-confluent sheets of white masses, some with central punctate hemorrhage, ranging in size from approximately 0.3 to 3 cm in the greatest dimension. These tumor masses replaced nearly 90% of the total liver volume. Histopathology (Figures 5 and 6) showed poorly differentiated squamous cell carcinoma secondary to metastasis. Review of prior pathology from the time of LC surgery (Figures 7 and 8) showed moderately differentiated squamous cell carcinoma, which was histopathologically consistent with the observed metastasis in the liver.

## 3. Discussion

Warfarin therapy has a narrow risk-to-benefit profile. Its pharmacokinetics is complex. The effective half-life of warfarin ranges from 20 to 60 hours, with a mean of about 40 hours. The maximum dose effect occurs up to 48 hr after administration of a single dose and persists for the next 5 days. The drug is completely absorbed after oral administration, and peak concentrations occur within 4 hours. The warfarin metabolism occurs mainly in the liver. It involves the cytochrome P450, and in particular, the CYP2C9 isoenzyme. Very little is excreted unchanged in the urine and the bile [6].

Supratherapeutic levels of anticoagulation with warfarin result from the administration of inappropriately high doses, altered protein binding, decreased vitamin K intake, reduced synthesis, or increased clearance of vitamin K-dependent clotting factors and the simultaneous use of other compounds that interfere with warfarin metabolism. Elderly patients can also exhibit an exaggerated response to warfarin, in part because they tend to store less vitamin K than younger people [7]. Therefore, it is not surprising that the most common complication of warfarin use is adverse bleeding [8].

Before concluding that warfarin toxicity is the responsible cause for coagulopathy, many other conditions need to be considered. Differential diagnosis of prolonged PT and PTT is numerous and can be divided into inherited and acquired. Inherited causes include prothrombin, fibrinogen, factor V, X, and combined factor deficiency. Acquired causes are mostly due to impaired synthesis, loss, or increased consumption or inhibition of coagulation factors. Impaired synthesis stems from vitamin K deficiency or hepatic disease. Massive bleeding may be responsible for the loss of coagulation factors, when the intravascular volume is replaced by crystalloids, colloids, and red blood cells without replacing coagulation factors. Disseminated Intravascular Coagulation (DIC) pathophysiology is explained by increased consumption of coagulation factors. Inhibition of coagulation factors is seen with presence of inhibitor antibodies to prothrombin, fibrinogen, factor V, X, or direct thrombin inhibitor or iatrogenic with use of vitamin K antagonists (warfarin) or with use of heparin or combined warfarin and heparin use. Warfarin is highly bound (approximately 97%) to plasma protein, mainly albumin. The high degree of protein binding is one of several mechanisms whereby other drugs interact with warfarin. Liver failure may be differentiated from vitamin K deficiency by measuring factor V, which is not vitamin K-dependent [9]. The presence of inhibiting antibodies can be confirmed by mixing studies. A diagnosis of DIC may be made using a simple scoring system based on platelet count, PT, D-dimer levels, and fibrinogen levels [10]. In the case described above, there was no evidence to suggest intentional overdose of warfarin, no use of compound that could have potentially increased the warfarin levels, and no prior history of inherited bleeding disorder. In our clinical practice, just as reported in Budnitz et al. [5], majority of coagulation abnormalities detected upon admission to our hospital are related to warfarin.

Our patient presented with severe warfarin induced coagulopathy. Results of autopsy revealed near complete infiltration of the liver with laryngeal cancer metastasis. The liver is the most common site for metastatic tumor deposits with evidence of hepatic metastasis in 36% of all patients who die from cancer [11]. Diffuse parenchymal metastasis is a rare pattern of liver metastasis. Watson reviewed the literature from the period 1868 to 1954 and reported 18 such cases [12]; Rowbotham and colleagues recognized 18 (0.44%) patients with fulminant hepatic failure (FHF) attributable to cancerous hepatic infiltration among 4020 hospital admissions [11]. The incidence of distant metastases in squamous cell carcinoma of head and neck approaches 20%–25%. The most common sites of metastases are lung (70%–75%), liver (17%–38%), and bone (23%–44%) [13]. Acute liver failure (ALF) secondary to diffuse metastatic infiltration of the liver is rare and has an extremely poor prognosis.

The mechanism of ALF in the setting of neoplastic infiltration is multifactorial. Massive cytokine release has been implicated as a cause of liver failure. Cytokine release can cause liver failure by damaging bile ducts both directly and via recruitment of effector cells, and by activation of leucocytes and hepatic sinusoidal cells, thus impeding hepatic sinusoidal microcirculation. Liver failure may also occur due to ischemia produced by tumor emboli compromising the portal venous circulation, or nonocclusive infarction of liver due to shock from other causes such as sepsis or cardiac dysfunction. The direct effect of tumor infiltration with replacement of hepatocytes is probably more important as a mechanism in nonhaematological malignancies. Indeed, FHF rarely develops in metastatic carcinomatosis in the absence of hypotension [14]. Our patient had normal LFTs four weeks prior to the development of ALF and then rapidly progressed. She has had infiltration of the liver over a period of time with no evidence of clinical or laboratory abnormalities, which then rapidly progressed to ALF as a result of further insult from hypotension and cytokine release likely secondary to sepsis or cardiac event.

Clinical presentation and laboratory findings of neoplastic infiltration of liver are vague and far from being pathognomonic. Hyperbilirubinemia may be the result of either hepatic parenchymal infiltration or extrahepatic biliary obstruction. It is well known that the increase in serum aminotransferases represents liver cell destruction and may be the only laboratory test indicating liver dysfunction prior to its clinical manifestation. But in our patient her LFTs were normal four weeks prior to admission. However, elevated serum LDH levels appear to correlate better with metastasis-related hepatic failure, since it is believed that elevated levels represent rapid tumor growth, by reflecting either the liver cell destruction process or an elevated production of LDH enzyme by neoplastic cells themselves. There are reports that correlate LDH serum levels with hepatic metastases from malignant melanoma, small cell lung cancer (SCLC) patients [15–18]. Extremely high serum LDH levels represent diffuse replacement of the liver parenchyma and are associated with a higher risk of development of FHF and a poor prognosis [19, 20]. Several authors suggest that an increase in serum LDH levels in cancer patients may prelude ALF [16, 21, 22]. These findings are well supported by the results of LFTs in our patient, who had extremely elevated serum LDH levels. Death is usually a direct consequence of the FHF, rather than the underlying malignancy. Acute warfarin toxicity on the stable dose of warfarin without any alternative cause was not a presentation in any of the reported case series. Because of the rapid progression of FHF, appropriate imaging procedures are usually difficult to perform. However, CT scan of the abdomen in our patient showed markedly enlarged liver with heterogeneity suggesting infiltrative process (Figure 2).

There have been case reports of liver metastasis-induced FHF from haematologic malignancies [23–25], breast cancer [26, 27], small cell carcinoma of lung [16, 28–30], colon cancer, urothelial cancer [31], and malignant melanoma [20, 22, 32–34]. Coagulopathy, on stable dose of warfarin, was not a presenting feature in any of these cases, but in our indexed case the main presentation was severe coagulopathy associated with ALF secondary to near complete infiltration of liver with metastatic disease. Unfortunately, the prognosis of patients with FHF resulting from malignant infiltration is dismal. The majority of patients do not survive shortly after the onset of liver failure [11, 26, 35]. According to a review by Allison et al., concerning 21 reported cases of ALF due to metastatic breast carcinoma, 18 cases died within 3 days to 7 months. Regrettably our patient died in less than 24 hours of admission to ICU.

## 4. Conclusion

Delayed distant metastasis is rare in head and neck cancer. ALF secondary to malignant infiltration of the liver due to delayed distant metastasis from laryngeal cancer was never reported in the literature to our best knowledge. Acute warfarin toxicity on stable dose of warfarin without any alternative cause is rare. Neoplastic infiltration of liver should be considered in the differential diagnosis when patients present with severe coagulopathy with ALF, and laboratory evidence of cellular destruction. Efforts must be made to determine the etiology of the disease, as it influences prognosis and prompt institution of specific therapies that might lead to recovery. Supportive care with close communication concerning end-of-life issues should be considered the standard of care in patients presenting with ALF secondary to solid tumor malignancies since the prognosis is invariably poor.

## Figures and Tables

**Table 1:** Patient's laboratory values during hospitalization until death.

Parameter	⟶					
	Hour (hr) 0	hr 6	hr 15	hr 18	hr 22	hr 30
PT (seconds)	>169	275.3	57.7	61.1	72	130.3
INR	Unrecordable	23.8	5.1	5.4	6.4	11.4
PTT (seconds)	92	66.8	44.3	46.7	54.1	98.9
Serum albumin (g/dL)	2.8		2.4	2.6	2.1	1.8
Alanine aminotransferase (unit/L)	420		1234	1201	1312	2179
Aspartate transaminase (unit/L)	973		2475	2344	2548	3681
Alkaline phosphatase (unit/L)	435		393	369	403	379
Total bilirubin (mg/dL)	1		1.4	1.5	1.3	1.3
Lactic acid level (mmoles/L)	13.3		6.6	17	18	15
LDH (unit/L)			8003			
Troponin (ng/mL)	0.21		0.32	0.48	0.88	1.58
CK (unit/L)	872		1059	1220	1179	1283
CK-MB (ng/mL)	40.42		44.01	53.75	66.06	72.28
CK-MB%	4.6		4.2	4.2	5.6	5.6
Haemoglobin (g/dL)	12		7.2	9.7	8.7	7.8
Bicarbonate (mEq/L)	10	13		5	6	3

**Table 2:** Warfarin dosing and INR prior to hospitalization.

Time frame	INR	Warfarin dose (mg)
2 weeks ago	2.8	4
2 months ago	2.4	4
6 months ago	2.2	4
1 year ago	2.9	4
1.5 year ago	3.7	5
